# Nationwide surveillance of carbapenem-resistant Gram-negative pathogens in the Lebanese environment

**DOI:** 10.1128/aem.01932-24

**Published:** 2025-06-10

**Authors:** Zahraa F. Samadi, Zeinab R. Hodroj, Ziad C. Jabbour, Hadi M. Hussein, Abdallah Kurdi, Dayana Shoukair, Ricardo F. Bitar, Hadi H. Chebaro, Jean Marie J. Al Semaani, Mohamad T. Al Hajjar, Housein H. Zeaiter, Lama Hamadeh, Rami Mahfouz, Lama H. Noueihed, Jad H. Hachem, Mahmoud I. Khalil, Rana El Hajj, Ghassan M. Matar, Antoine G. Abou Fayad

**Affiliations:** 1 Department of Experimental pathology Immunology & microbiology, Faculty of Medicine, American University of Beirut 66978 , Beirut, Lebanon; 2 Center for Infectious Diseases Research, American University of Beirut 11238, Beirut, Lebanon; 3 World Health Organization (WHO) Collaborating Center for Reference and Research on Bacterial Pathogens, Beirut, Lebanon; 4 Department of Biological Sciences, Faculty of Science, Beirut Arab University 67025, Beirut, Lebanon; 5 Department of Biochemistry and Molecular Genetics, Faculty of Medicine, American University of Beirut 66978 , Beirut, Lebanon; 6 Department of Pathology and Laboratory Medicine, American University of Beirut Medical Center 66984, Beirut, Lebanon; 7 Pillar Genomics Laboratory, American University of Beirut 11238, Beirut, Lebanon; 8 Department of Life and Earth Sciences, Faculty of Science, Lebanese University 247640, Beirut, Lebanon; 9 Molecular Biology Unit, Department of Zoology, Faculty of Science, Alexandria University 534910, Alexandria, Egypt; Washington University in St. Louis, St. Louis, Missouri, USA

**Keywords:** environmental surveillance, ESKAPE pathogens, Gram-negative, antimicrobial resistance, carbapenem resistance, whole-genome sequencing, Lebanon

## Abstract

Gram-negative ESKAPE pathogens with carbapenem resistance pose a significant health threat. Despite extensive research on the spread of these pathogens within Lebanese hospital settings, their emergence in environmental settings remains understudied. This study aimed to explore the environmental spread of carbapenem resistance among Gram-negative bacteria isolated from environmental samples in nine districts across Lebanon. A total of 250 samples were collected from wild animals, sewage, water, and soil between June 2022 and September 2023. Samples were streaked on MacConkey agar plates supplemented with 2 mg/L meropenem. Bacterial species were identified primarily using API20E. Antimicrobial susceptibility profiles were determined by the disk diffusion method and the Vitek 2 compact system. Meropenem-resistant Gram-negative bacteria were further characterized by whole-genome sequencing, and each of the bacterial species, sequence types, resistance genes, and plasmids was detected by sequence data analysis. We successfully isolated 130 carbapenem-resistant isolates from various samples, 67 of which belonged to the ESKAPE pathogens list and showed a multidrug-resistant (MDR) profile. The distribution of the latter was as follows: *Escherichia coli* (65.67%), *Acinetobacter baumannii* (16.42%), *Pseudomonas aeruginosa* (11.94%), and *Klebsiella pneumoniae* (5.97%). Several carbapenem resistance genes were detected, with a prevalence of *bla*NDM-5 in *Escherichia coli* and *Klebsiella pneumoniae*, *bla*IMP-1 and mexAB-OprM efflux pumps in *Pseudomonas aeruginosa*, and *bla*OXA-23 in *Acinetobacter baumannii*. Our findings revealed a widespread distribution of carbapenem-resistant ESKAPE bacteria in Lebanon, underscoring the significant public health risk posed by these pathogens. This highlights the urgent need to address the dissemination of antibiotic resistance in Lebanese environmental settings.

**IMPORTANCE:**

The emergence of antimicrobial resistance (AMR) extremely burdens public health and increases morbid and mortal threats in Lebanon. While the majority of the studies in our country target antimicrobial resistance in clinical settings, fewer studies focus on antimicrobial resistance dissemination in the environment. The significance of our research is that it sheds light on the environment as a less explored yet equally crucial sector in the spread of AMR. Here, we isolated carbapenemase-producing bacteria (*Escherichia coli*, *Klebsiella pneumoniae*, *Pseudomonas aeruginosa*, and *Acinetobacter baumannii*) that were categorized as multidrug resistant (MDR) from diverse environmental sources in multiple provinces across Lebanon. The finding of carbapenem-resistant bacteria carrying plasmids represents a potential risk due to the possible spread of resistance genes via horizontal gene transfer across the environment and hospital settings. This highly recommends the implementation of regular surveillance to monitor the spread of antimicrobial resistance among environmental bacteria, which consequently leads to its spread within communities and thus poses a great threat to human health.

## INTRODUCTION

Antimicrobial resistance is a critical global concern recognized by the World Health Organization (WHO), where microorganisms lose sensitivity to antimicrobial drugs (1). The spread of antimicrobial-resistant bacteria and antibiotic resistance genes (ARGs) poses a significant human threat (2). The primary cause of antimicrobial resistance (AMR) is the overuse of antibiotics in clinical, veterinary, and agricultural settings, and the migration of infected individuals and animals (3). The AMR crisis is projected to result in approximately 10 million deaths per year by 2050 (4). Addressing this issue necessitates recognizing the interconnection between humans, animals, and the environment (5). The global spread of antibiotic resistance underscores the critical need for activating AMR surveillance systems, which primarily rely on collecting laboratory data at either local or national levels (6). These systems provide valuable information on the epidemiology of AMR, aiding in the development of effective strategies to reduce its emergence and spread (7). However, current surveillance programs primarily focus on AMR in livestock and isolates from human clinical cases, often omitting environmental perspectives, including wildlife (8). Additionally, routine AMR surveillance is lacking in many low- and middle-income countries, including Lebanon (7). Thus, incorporating environmental surveillance at the national level could enhance our understanding of AMR circulation within human populations, thereby improving existing human clinical surveillance systems (8).

Carbapenem, a β-lactam antibiotic, serves as one of the last-resort antibiotics for human treatment. Carbapenem resistance can emerge as a consequence of several mechanisms, including active efflux pumps, porin loss, and, more importantly, the production of the carbapenemase enzyme (9). Carbapenemases are divided according to the Ambler classification system into class A serine β-lactamases, class B metallo-β-lactamases with subclasses: B1, B2, and B3, and class D oxacillinases (10). In 2017, the WHO published a list of antimicrobial-resistant pathogens prioritized into critical, high, and medium (11). Recently, the WHO Bacterial Priority Pathogen List 2024 includes the following bacteria among critical pathogens, multidrug-resistant (MDR) Gram-negative ESKAPE pathogens (carbapenem-resistant *Acinetobacter baumannii* [CRAB], third-generation cephalosporin-resistant Enterobacterales, and carbapenem-resistant Enterobacterales [CRE]), and among high priority carbapenem-resistant *Pseudomonas aeruginosa* (CRPA) associated with hospital-acquired infections such as pneumonia, meningitis, bloodstream, and urinary tract infections (11). AMR is the leading cause of the significant increase in morbidity and mortality worldwide and particularly in Lebanon, where the country faces a multitude of challenges, including social, health, and economic crises (12). Alarming instances of carbapenem resistance have been identified in Gram-negative bacteria in Lebanon (13, 14). Although existing studies have focused on clinical settings (15), limited surveillance has been conducted to examine carbapenem-resistant Gram-negative bacteria in the environment and animals. Since data on the presence and dissemination of carbapenem-resistant bacteria in Lebanon is scarce, this study aims to address the prevalence of carbapenem resistance in Gram-negative bacteria across the nation.

## RESULTS

### Collection and identification of bacterial isolates

**Fig 1:**
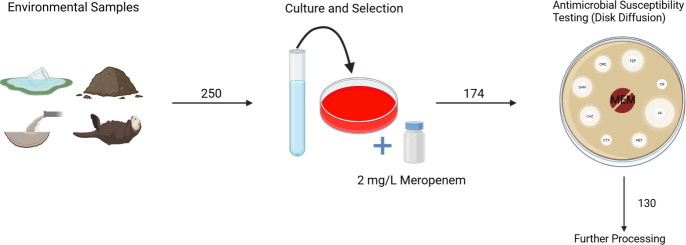
Collection and filtration of samples.

Between June 2022 and September 2023, a total of 250 environmental samples were collected across Lebanon, out of which 174 isolates were recovered on MacConkey agar supplied with 2 mg/L meropenem. Those were mainly recovered from several environmental samples, including water sources (*n* = 97/174, 55.74%), sewage (*n* = 39/174, 22.41%), animal (*n* = 29/174, 16.66%), and soil (*n* = 9/174, 5.17%). Out of 174, only 130 isolates showed resistance against meropenem according to the Kirby-Bauer disk diffusion method. Figure 1 summarizes the different collection and filtering steps.

**Fig 2:**
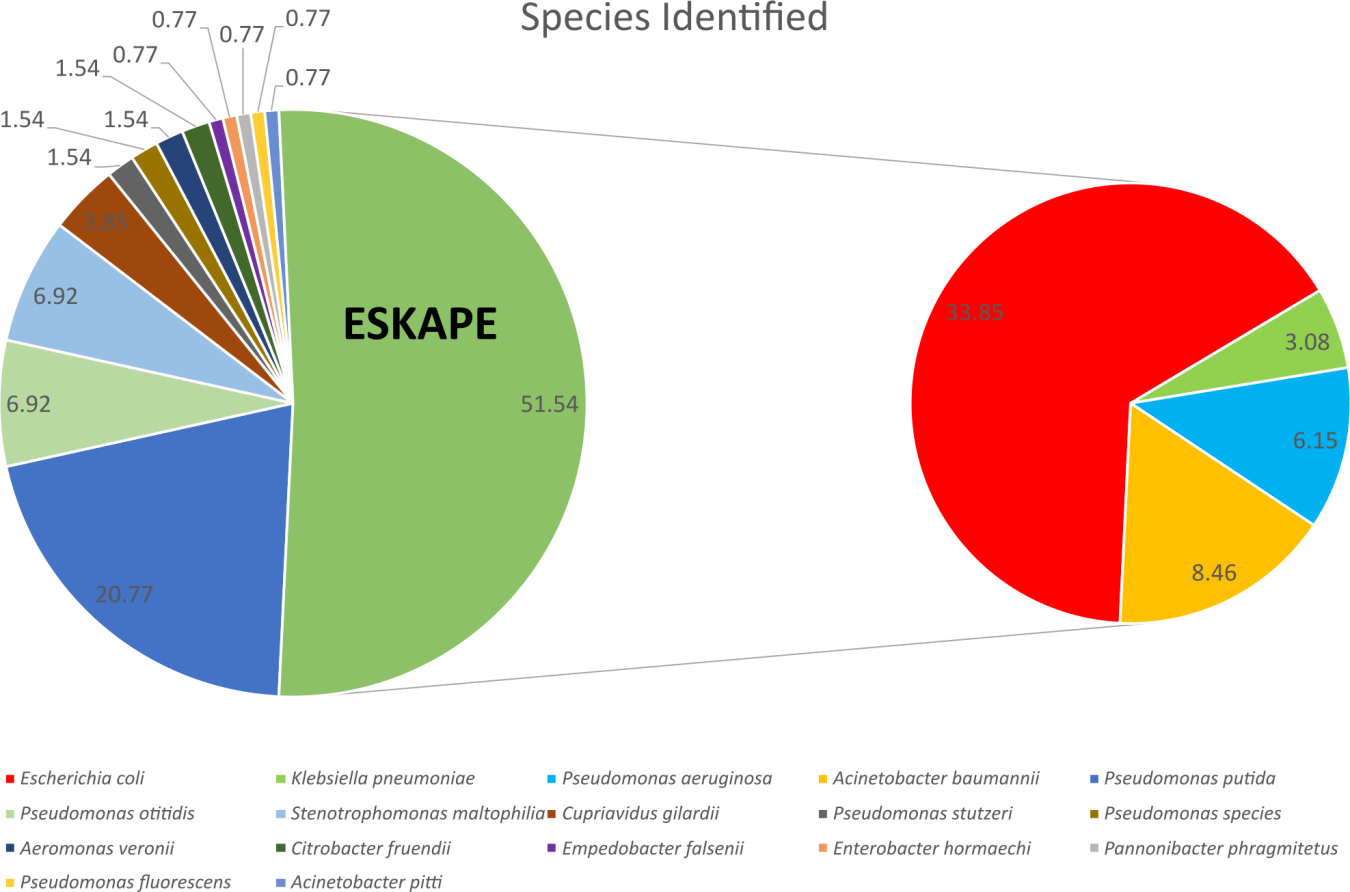
Identification of meropenem-resistant isolates.

In total, identification of (*n* = 48/130, 37.70%) CRE including 33.85% of *Escherichia coli* (*n* = 44/130), and 3.08% of *Klebsiella pneumoniae* (*n* = 4/130), CRPA (*n* = 8/130, 6.15%), and CRAB (*n* = 11/130, 8.46%). Other species were isolated, including but not limited to *Pseudomonas* spp. (*n* = 36/130, 27.69%), *Cupriavidus gilardii* (*n* = 5/130, 3.84%), *Aeromonas veronii* (*n* = 2/130, 1.53%), and *Empedobacter falsenii* (*n* = 1/130, 0.76%). Figure 2 represents all meropenem-resistant isolates.

### Geographical distribution of carbapenem-resistant species

**Fig 3:**
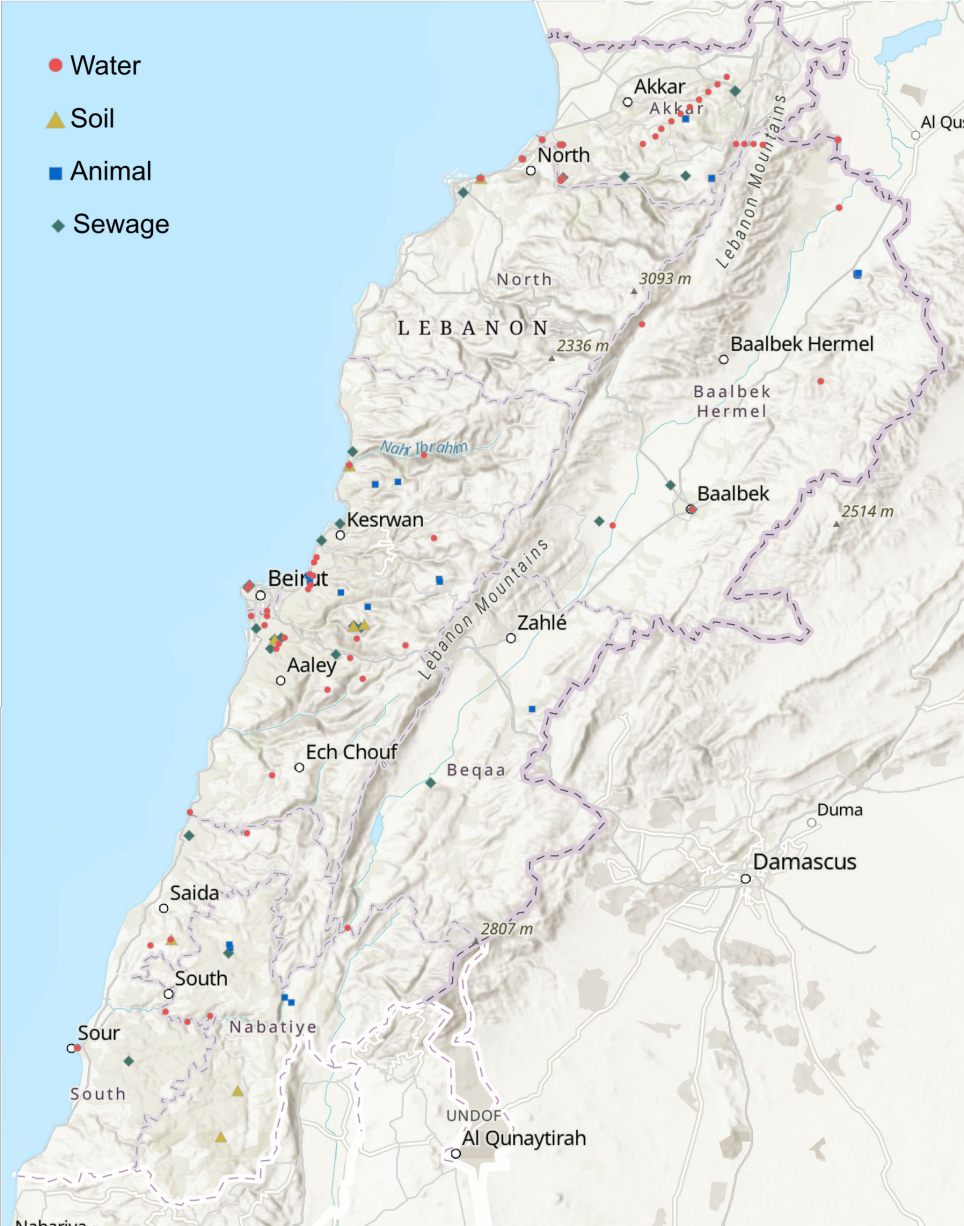
Geographical distribution of carbapenem-resistant Gram-negative bacteria across Lebanon. The environmental samples were sewage, animal, water, and soil (generated using ArcGIS Online, https://www.arcgis.com).

A total of 130 carbapenem-resistant isolates from different environments were collected from the nine provinces: Akkar, Baalbek-Hermel, Beirut, Beqaa, Keserwan-Jbeil, Mount Lebanon, Nabatieh, North, and South (Fig. 3).

### Antimicrobial susceptibility testing

Disk diffusion assay was done on all samples received. All samples were subjected to five different antibiotics from different classes, and resistance was analyzed based on CLSI M100Ed33 guidelines (16). Results revealed (*n* = 130/174, 74.71%) resistance to meropenem, lower resistance rates were observed against other antimicrobial agents as follows: (*n* = 81/174, 46.55%) to ceftazidime, (*n* = 73/174, 41.95%) to ciprofloxacin, (*n* = 63/174, 36.21%) to aztreonam, and (*n* = 38/174, 21.84%) to gentamicin. Accordingly, out of 174 isolates, 43.10% can be categorized as MDR bacteria since they show resistance to at least three antimicrobial agents from three different classes. Isolates that exhibited resistance to meropenem were further characterized by whole-genome sequencing.

### Distribution of carbapenem-resistant isolates by species

Table 1 summarizes the different phenotypic and genotypic AMR profiles for the four major species highlighted in this study. Isolates belonging to the ESKAPE group have been further tested against more antimicrobial agents for a clearer understanding of their resistance profiles.

**TABLE 1:** Phenotypic and genotypic profiles of *Escherichia coli*, *Klebsiella pneumoniae, Pseudomonas aeruginosa*, and *Acinetobacter baumannii*^*a*^

Isolate ID	ST	Antimicrobials and susceptibility																Resistance genes				
		β-lactams	β-lactam +β-lactamase inhibitors		Mono-bactam	Cephalosporins				Carbapenems		Aminoglycosides		Fluoro-quinolones	Tetra-cyclines	Nitro-furans	Sulfonamides	Carbapenem	Beta-lactams	Fluoro-quinolones	Others	
						1st	3rd		4th													
		AM	TZP	SAM	ATM	CZ	CRO	CAZ	FEP	MEM	ETP	AN	GMN	CIP	TGC	FT	SXT				Sulfonamides	Macrolides
ECOL_194	361	R	R	R	R	R	R	R	R	R	R	S	S	R	S	S	R	*bla*_*NDM-5*_	*ampC*	*gyrA, parC*	*sul1*	*mphA, Mrx*
ECOL_195	361	R	R	R	R	R	R	R	R	R	R	S	S	R	S	S	R	*bla*_*NDM-5*_	*ampC*	*gyrA, parC*	*sul1*	*mphA, Mrx*
ECOL_196	405	R	R	R	S	R	R	R	R	R	R	S	S	R	S	S	S	*bla*_*NDM-5*_	*bla*_*TEM-1*_	*gyrA, parC*	–^*b*^	*mphA, Mrx*
ECOL_197	405	R	R	R	R	R	R	R	R	R	R	S	S	R	S	S	R	*bla*_*NDM-5*_	*bla*_*OXA-1*_ *, bla*_*CTX-M-15*_ *, bla*_*TEM-1*_	*gyrA, parC*	*sul1*	*mphA, Mrx*
ECOL_198	648	R	R	R	R	R	R	R	R	R	R	S	R	R	S	R	R	*bla*_*NDM-5*_	*bla*_*OXA-1*_ *, bla*_*CTX-M-15*_ *, bla*_*TEM-1*_	*gyrA, parC*	*sul1*	*mphA, Mrx*
ECOL_199	405	R	R	R	R	R	R	R	R	R	R	S	S	R	S	S	R	*bla*_*NDM-5*_	*bla*_*CTX-M-15*_	*gyrA, parC*	*sul1*	*–*
ECOL_200	361	R	R	R	R	R	R	R	R	R	R	S	S	R	S	R	R	*bla*_*NDM-5*_	*ampC*	*gyrA, parC*	*sul1*	*mphA, Mrx*
ECOL_201	405	R	R	R	R	R	R	R	R	R	R	S	S	R	S	S	R	*bla*_*NDM-5*_	*bla*_*OXA-1*_ *, bla*_*CTX-M-15*_ *, bla*_*TEM-1*_	*gyrA, parC*	*sul1*	–
ECOL_202	405	R	R	R	R	R	R	R	R	R	R	S	S	R	S	S	R	*bla*_*NDM-5*_	*bla*_*OXA-1*_ *, bla*_*CTX-M-15*_ *, bla*_*TEM-1*_	*gyrA, parC*	*sul1*	*mphA, Mrx*
ECOL_203	405	R	R	R	R	R	R	S	S	R	R	S	S	S	S	R	R	*bla*_*NDM-5*_	*bla*_*OXA-1*_ *, bla*_*CTX-M-15*_ *, bla*_*TEM-1*_	–	*sul1*	*mphA, Mrx*
ECOL_205	361	R	R	R	R	R	R	R	R	R	R	S	S	R	S	S	R	*bla*_*NDM-5*_	*bla*_*OXA-1*_ *, ampC*	*gyrA, parC*	*sul1*	*mphA, Mrx*
ECOL_206	405	R	R	R	R	R	R	R	R	R	R	S	S	R	S	S	R	*bla*_*NDM-5*_	*bla*_*OXA-1*_	*gyrA, parC*	*sul1*	*mphA, Mrx*
ECOL_207	405	R	R	R	R	R	R	R	R	R	R	S	S	R	S	S	R	*bla*_*NDM-5*_	–	*gyrA, parC*	*sul1*	*mphA, Mrx*
ECOL_208	2083	R	R	R	R	R	R	R	R	R	R	S	S	R	S	S	R	*bla*_*NDM-5*_	*bla*_*TEM-1*_	*gyrA, parC*	*sul1*	*mphA, Mrx*
ECOL_209	361	R	R	R	R	R	R	R	R	R	R	S	S	R	S	S	R	*bla*_*NDM-5*_	*bla*_*OXA-1*_ *, ampC*	*gyrA, parC*	*sul1*	*mphA, Mrx*
ECOL_210	167	R	S	R	S	R	S	S	S	R	R	S	S	R	R	R	R	*bla*_*NDM-5*_	*bla*_*CTX-M-15*_ *, bla*_*TEM-169*_	*gyrA, parC*	*sul1*	*mphA, Mrx*
ECOL_211	405	R	R	R	R	R	R	R	R	R	R	S	S	R	S	S	R	*bla*_*NDM-5*_	*bla*_*OXA-1*_ *, bla*_*CTX-M-15*_ *, bla*_*TEM-1*_	*gyrA, parC*	*sul1*	*mphA, Mrx*
ECOL_212	-	R	R	R	R	R	R	R	R	R	R	S	S	R	S	S	R	–	–	*gyrA,*	*sul1*	*–*
ECOL_213	2083	R	R	R	R	R	R	R	R	R	R	S	S	R	S	S	R	*bla*_*NDM-5*_	*bla*_*TEM-1*_	*gyrA, parC*	*sul1*	*mphA, Mrx*
ECOL_216	361	R	R	R	R	R	R	R	R	R	R	S	S	R	S	S	R	*bla*_*NDM-5*_	*ampC*	*gyrA, parC*	*sul1*	*mphA, Mrx*
ECOL_217	361	R	R	R	R	R	R	R	R	R	R	S	S	R	S	S	R	*bla*_*NDM-5*_	*ampC*	*gyrA, parC*	*sul1*	*mphA, Mrx*
ECOL_218	167	R	R	R	R	R	R	R	R	R	R	S	S	R	S	S	R	*bla*_*NDM-5*_	*bla*_*OXA-1*_ *, bla*_*CTX-M-15*_	*gyrA, parC*	*sul1*	*mphA, Mrx*
ECOL_219	361	R	R	R	R	R	R	R	R	R	R	S	S	R	S	S	R	*bla*_*NDM-5*_	*bla*_*TEM-1*_ *, ampC*	*gyrA, parC*	*sul1*	*mphA, Mrx*
ECOL_220	361	R	R	R	R	R	R	R	R	R	R	R	R	S	S	R	S	*bla*_*NDM-5*_	*bla*_*OXA-1*_ *, ampC*	*–*	*–*	*mphA, Mrx*
ECOL_221	90	R	R	R	R	R	R	R	R	R	R	S	S	R	S	S	R	*bla*_*NDM-5*_	*–*	*gyrA, parC*	*sul1*	*mphA, Mrx*
ECOL_222	10	R	R	R	R	R	R	R	R	R	R	R	R	R	R	R	R	*–*	*bla*_*TEM-1*_ *, ampC*	*–*	*sul1*	*mphA, Mrx*
ECOL_223	361	R	R	R	R	R	R	R	R	R	R	S	S	R	S	S	R	*bla*_*NDM-5*_	*bla*_*CTX-M-15*_	*gyrA, parC*	*sul1*	*mphA, Mrx*
ECOL_225	167	R	R	R	R	R	R	R	R	R	R	S	S	R	S	S	R	*bla*_*NDM-5*_	*bla*_*CTX-M-15*_ *, bla*_*TEM-169*_	*gyrA, parC*	*sul1*	*mphA, Mrx*
ECOL_226	167	R	R	R	R	R	R	R	R	R	R	S	S	R	S	S	R	*bla*_*NDM-5*_	*bla*_*CTX-M-15*_ *, bla*_*TEM-169*_	*gyrA, parC*	*sul1*	*mphA, Mrx*
ECOL_227	-	R	R	R	R	R	R	R	R	R	R	S	S	R	S	R	R	*bla*_*PST-2*_	*–*	*–*	*sul1*	*–*
ECOL_228	405	R	R	R	R	R	R	R	R	R	R	S	S	R	S	S	R	*bla*_*NDM-5*_	*–*	*gyrA, parC*	*sul1*	*mphA, Mrx*
ECOL_229	361	R	R	R	R	R	R	R	R	R	R	S	S	R	S	S	R	*bla*_*NDM-5*_	*bla*_*OXA-1*_ *, ampC*	*gyrA, parC*	*sul1*	*mphA, Mrx*
ECOL_230	361	R	R	R	R	R	R	R	R	R	R	S	S	R	S	S	R	*bla*_*NDM-5*_	*ampC*	*gyrA, parC*	*sul1*	*mphA, Mrx*
ECOL_231	405	R	R	R	R	R	R	R	R	R	R	S	S	R	S	S	R	*bla*_*NDM-5*_	*bla*_*OXA-1*_ *, bla*_*CTX-M-15*_ *, bla*_*TEM-1*_	*gyrA, parC*	*sul1*	*mphA, Mrx*
ECOL_232	167	R	S	R	R	R	R	R	S	R	R	S	S	R	S	R	R	*bla*_*NDM-5*_ *, bla*_*PAM-1*_	*bla*_*CTX-M-15*_ *, bla*_*TEM-169*_	*gyrA, parC*	*sul1*	*mphA, Mrx*
ECOL_233	361	R	R	R	R	R	R	R	R	R	R	S	S	R	S	S	R	*bla*_*NDM-5*_	*ampC*	*gyrA, parC*	*sul1*	*mphA, Mrx*
ECOL_234	405	R	R	R	R	R	R	R	R	R	R	S	S	R	S	S	R	*bla*_*NDM-5*_	*–*	*gyrA, parC*	*sul1*	*mphA, Mrx*
ECOL_235	2083	R	R	R	R	R	R	R	R	R	R	S	S	R	S	S	R	*bla*_*NDM-5*_	*bla*_*TEM-1*_	*gyrA, parC*	*sul1*	*mphA, Mrx*
ECOL_236	361	R	R	R	R	R	R	R	R	R	R	S	S	R	S	S	R	*bla*_*NDM-5*_	*ampC*	*gyrA, parC*	*sul1*	*mphA, Mrx*
ECOL_237	405	R	R	R	R	R	R	R	R	R	R	S	S	R	S	S	R	bla_NDM-5_	*–*	gyrA, parC	sul1	mphA, Mrx
ECOL_238	10	R	R	R	R	R	R	R	R	R	R	R	R	S	R	R	S	*–*	*bla*_*TEM-1*_ *, ampC*	*–*	*–*	*–*
ECOL_239	167	R	R	R	R	R	R	R	R	R	R	S	S	R	S	R	R	*bla*_*NDM-5*_	*bla*_*OXA-1*_ *, bla*_*CTX-M-15*_	*gyrA, parC*	*sul1*	*mphA, Mrx*
ECOL_240	361	R	R	R	R	R	R	R	R	R	R	S	S	R	S	S	R	*bla*_*NDM-5*_	*bla*_*CTX-M-15*_	*gyrA, parC*	*sul1*	*mphA, Mrx*
ECOL_241	167	R	R	R	R	R	R	R	R	R	R	S	R	R	S	R	R	*bla*_*NDM-5*_	*–*	*gyrA,*	*sul1*	*–*
KLB_111	16		R	R	R	R	R	R	R	R	R	R	R	R	S	R	R	*bla*_*NDM-5*_ *, bla*_*OXA-181*_	*bla*_*SHV-11*_ *, bla*_*SHV-173*_ *, bla*_*CTX-M-15*_ *, bla*_*TEM-1*_	*gyrA*	*sul1*	*mphA, Mrx*
KLB_112	147		R	R	R	R	R	R	R	R	R	R	R	R	R	R	R	*bla*_*NDM-5*_	*bla*_*SHV-11*_ *, bla*_*CTX-M-15*_ *, bla*_*TEM-1*_	*gyrA, parC*	*sul1*	*mphA, Mrx*
KLB_113	15		R	R	R	R	R	R	R	R	R	R	R	R	R	R	R	*bla*_*NDM-1*_	*bla*_*OXA-1*_ *, bla*_*SHV-28*_ *, bla*_*CTX-M-15*_ *, bla*_*TEM-1*_	*gyrA, parC*	*sul1, sul2*	*mphA, Mrx*
KLB_114	147		R	R	R	R	R	R	R	R	R	S	S	R	R	R	R	*bla*_*NDM-1*_	*bla*_*OXA-1*_ *, bla*_*OXA-9*_ *, bla*_*SHV-11*_ *, bla*_*CTX-M-15*_ *, bla*_*TEM-1*_	*gyrA, parC*	*sul1*	*–*
ACN_424	2		R	R			R	R	R	R			R	R	R		S	*bla*_*OXA-23*_	*bla*_*OXA-66*_ *, bla*_*ADC-73*_	*gyrA*	*–*	*mphE, msrE*
ACN_425	2		R	R			R	R	R	R			R	R	R		S	*bla*_*OXA-23*_	*bla*_*OXA-66*_ *, bla*_*ADC-73*_	*gyrA*	*–*	*mphE, msrE*
ACN_426	2		R	R			R	R	R	R			R	R	R		S	*bla*_*OXA-23*_	*bla*_*OXA-66*_ *, bla*_*ADC-73*_	*gyrA*	*–*	*mphE, msrE*
ACN_427	2		R	R			R	R	R	R			R	R	R		S	bla_OXA-23_	bla_OXA-66_, bla_ADC-73_	gyrA	*–*	mphE, msrE
ACN_228	2		R	R			R	R	R	R			R	R	R		S	*bla*_*OXA-23*_	*bla*_*OXA-66*_ *, bla*_*ADC-73*_	*gyrA*	*–*	*mphE, msrE*
ACN_429	2		R	R			R	R	R	R			R	R	R		S	*bla*_*OXA-23*_	*bla*_*OXA-66*_ *, bla*_*ADC-73*_	*gyrA*	*–*	*mphE, msrE*
ACN_430	2		R	R			R	R	R	R			R	R	R		S	*bla*_*OXA-23*_	*bla*_*OXA-66*_ *, bla*_*ADC-73*_	*gyrA*	*–*	*mphE, msrE*
ACN_431	2		R	R			R	R	R	R			R	R	R		S	*bla*_*OXA-23*_	*bla*_*OXA-66*_ *, bla*_*ADC-73*_	*gyrA*	*–*	*mphE, msrE*
ACN_432	2		R	R			R	R	R	R			R	R	R		S	*bla*_*OXA-23*_	*bla*_*OXA-66*_ *, bla*_*ADC-73*_	*gyrA*	*–*	*mphE, msrE*
ACN_435	968		R	R			R	R	R	S			S	S	S		S	*–*	*bla*_*OXA-531*_ *, bla*_*ADC-98*_	*–*	*–*	*–*
ACN_436	2		R	R			R	R	R	R			R	R	R		S	*bla*_*OXA-23*_	*bla*_*OXA-66*_ *, bla*_*ADC-73*_	*gyrA, ParC*	*–*	*mphE*
PSA_701	244		R					R	R	R		S	R	R				*mexA/B OprM*	*bla*_*OXA-847*_	*–*	*sul1*	*–*
PSA_702	1182		S					S	S	R		S	S	R				*mexA/B OprM*	*bla*_*OXA-851*_	*gyrA*	*–*	*–*
PSA_703	1182		S					R	R	R		S	S	R				*mexA/B OprM*	*bla*_*OXA-851*_	*gyrA*	*–*	*–*
PSA_704	1182		S					S	S	R		S	S	R				*mexA/B OprM*	*bla*_*OXA-851*_	*gyrA*	*–*	*–*
PSA_705	1182		R					R	R	R		S	S	R				*mexA/B OprM*	*bla*_*OXA-851*_	*gyrA*	*–*	*–*
PSA_706	1182		S					R	R	R		S	R	R				*mexA/B OprM*	*bla*_*OXA-851*_	*gyrA*	*–*	*–*
PSA_707	244		R					R	R	R		S	S	R				*mexA/B OprM*	*bla*_*OXA-851*_	*–*	*–*	*–*
PSA_708	357		S					R	R	R		S	R	R				*bla*_*IMP-1,*_ *mexA/B OprM*	*bla*_*OXA-846*_ *, bla*_*OXA-10*_ *, bla*_*VEB-9*_	*gyrA, qnrVC1*	*sul1*	*–*
Legend:		R	Resistant					S	Susceptible			Empty		Not Evaluated								

^*a*^ AM, Ampicillin; TZP, Piperacillin/Tazobactam; SAM, Ampicillin/Sulbactam; ATM, Aztreonam; CZ, Cefazolin; CRO, Ceftriaxone; CAZ, Ceftazidime; FEP, Cefepime; MEM, Meropenem; ETP, Ertapenem; AN, Amikacin; GMN, Gentamicin; CIP, Ciprofloxacin; TGC, Tigecycline; FT, Nitrofurantoin; and SXT, Sulfamethoxazole/Trimethoprim; ESBL, extended-spectrum β-lactamase; S, susceptible; R, resistant; empty, not evaluated.

^*b*^ – signifies the absence of genes.

### ESKAPE pathogens

#### Antimicrobial resistance phenotypic profile

The detailed antimicrobial resistance profiles of all ESKAPE pathogens are summarized in Table 1. Briefly, all isolates exhibited resistance to beta-lactam antibiotics, first and second-generation cephalosporins, ceftriaxone, and meropenem. Resistance rates varied across species, with major differences in susceptibility against gentamicin, amikacin, and tigecycline. Therefore, all *Escherichia coli*, *Klebsiella pneumoniae*, *Pseudomonas aeruginosa,* and *Acinetobacter baumannii* isolates can be characterized as MDR.

#### Antimicrobial resistance genotypic profile

##### Carbapenem-resistance

Carbapenem-resistance mechanisms were observed among all ESKAPE pathogens, varying among species. The metallo-beta-lactamase (MBL) *bla*_NDM_ was prevalent in *Klebsiella pneumoniae* and *Escherichia coli* isolates (*n* = 4/4, 100% and *n* = 40/44, 90.91%, respectively). On the other hand, the carbapenem resistance mechanism in *Acinetobacter baumannii* was predominantly mediated by the co-expression of the *bla*_OXA_-type carbapenemases *bla*_OXA-23_ and *bla*_OXA-66_ along with AdeIJK efflux pumps (*n* = 10/11, 90.9%). Moreover, carbapenem resistance in *Pseudomonas aeruginosa* was exhibited through the MexA/B-OprM efflux pump system, a common resistance mechanism in CRPA (*n* = 8/8, 100%). Other noteworthy mechanisms of carbapenem resistance are MBL *bla*_IMP-1_ in CRPA (*n* = 1/8, 12.5%), OXA-58-like *bla*_OXA-531_ in CRAB (*n* = 1/1, 8.2%), and the MBL *bla*_PST-2_ (*n* = 1/44, 2.27%), in *E. coli*.

##### Other resistance

Other resistance mechanisms were observed among all isolates. The extended-spectrum beta-lactamases (ESBLs) *bla*_TEM_, *bla*_CTX-M-15_, and *bla*_OXA-1_ were the most commonly isolated in CRE isolates, *bla*_OXA-847_ and *bla*_OXA-851_ from *P. aeruginosa,* conferring resistance to most beta-lactam antibiotics. Moreover, cephalosporin resistance was mediated by a wide variety of genes, differing among species. These include *ampC* in *E. coli*, *bla*_ADC-73_ in *A. baumannii*, and *bla*_PDC_ in *P. aeruginosa*. In addition, *mphA* and *Mrx* genes were present in CRE isolates as mechanisms for macrolide resistance, and *mphE* and *msrE* in *A. baumannii*. Conversely, sulfonamide resistance was observed by all ESKAPE pathogens by the expression of the *sul1* gene, except for *A. baumannii* isolates, which all showed sensitivity to sulfonamides. Furthermore, fluoroquinolone resistance was homogenous among all species expressing *gyrA* mutation and/or *parC* as the main mechanism of resistance.

### Multilocus-sequence typing and plasmid typing

Using the multilocus-sequence typing (MLST) tool, a wide variety of sequence types were uncovered among species. A variety of relevant sequence types (ST) were identified among *E. coli* isolates, with predominance of ST361 (34.09%) and ST405 (29.54%), other notable STs are ST167 (15.91%), ST2083 (6.82%), and with smaller proportions for ST10, ST648, and ST90. A lesser variety of sequence types was observed for *P. aeruginosa* and *K. pneumoniae,* with three sequence types for each: ST1182 (62.5%), ST244, ST357, and ST147 (50%), ST15, ST16, respectively. In contrast, no variety of STs was observed among *A. baumannii* isolates, with predominance of ST2 (83.3%) and only one isolate of ST968.

Plasmid typing was done using PlasmidFinder. The full data on plasmid typing can be found in Table S1. In brief, Inc plasmids were the most prevalent across species. The most common Inc types included IncFII and IncFIA, which were found in *Escherichia coli* (90.91% and 84.91% of isolates, respectively), *Klebsiella pneumoniae* (present in all isolates), and *Pseudomonas aeruginosa* (in one isolate). Other Inc types observed included IncY (38.64% in *E. coli*), IncI1, IncI2, IncQ1, IncX1, and IncX3 (detected in lower frequencies, predominantly in *E. coli*). Col plasmids were also commonly found, especially Col(pHAD28), which was present in *Escherichia coli* (25%) and *Klebsiella pneumoniae* (75%). Other Col plasmids, such as Col(MG828) and Col(BS512), were less frequently detected. In addition, unique plasmid combinations certain isolates harbored multiple plasmid types, such as those from *E. coli* [e.g., IncFII + IncFIA + Col(MG828)] and *Klebsiella pneumoniae* [e.g., IncFIB + IncFII + Col(pHAD28)]. No plasmids were found in *A. baumannii* isolates.

### Other pathogens

Other Enterobacterales and non-Enterobacterales are shown in Table S2. The member of Enterobacterales, *E. hormaechi*, harbored *bla*_NDM-1_ along with the β-lactamase genes *bla*_OXA-1_ and *bla*_ACT-20_. The replicon types, IncFII(pKPX1) and IncF(repB(R1701)), were detected. In the case of *E. falsenii*, a non-Enterobacterales member, resistome analysis revealed the presence of the metallo-β-lactamase *bla*_EBR-4_. Additionally, *A. veronii* isolates exhibited the class B2 metallo-β-lactamase, *cphA3,* conferring intrinsic resistance to carbapenem along with the β-lactamase gene *bla*_OXA-912_ and ESBL gene *bla*_PER-3_. The assigned sequence type was ST2182, and only one plasmid, IncU, was detected. Also, *C. gilardii* (*n* = 2/5, 40%) co-harbored *mcr5.1* encoding colistin resistance and the β-lactamase gene, *bla*_OXA-837_. Most *Pseudomonas otitidis* (*n* = 8/9, 88.8%) carried the *bla*_POM-1_ gene, except for one isolate that harbored *bla*_POM-2_. Two *Pseudomonas stutzeri* isolates expressed the metallo-β-lactamase gene, *bla*_PST-2_ conferring resistance to carbapenems. Additionally, *Stenotrophomonas maltophilia* (*n* = 9/9, 100%) harbored *bla*_L1_ as an intrinsic mechanism of resistance to carbapenems.

### Geographical distribution of carbapenem resistance genes

**Fig 4:**
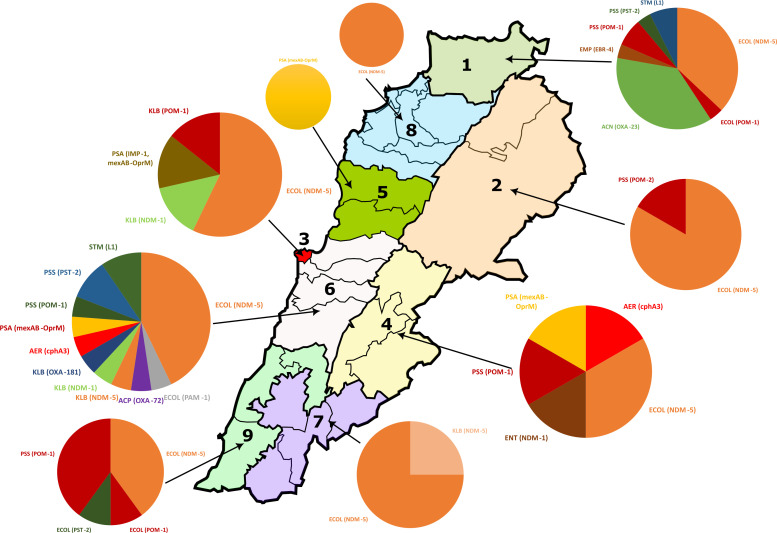
Map showing the geographical distribution of carbapenem resistance genes among different provinces. The governorate is represented as follows: 1—Akkar, 2—Baalbek-Hermel, 3—Beirut, 4—Beqaa, 5—Keserwan-Jbeil, 6—Mount Lebanon, 7—Nabatieh, 8—North, and 9—South. ECOL, *Escherichia coli*; KLB, *Klebsiella pneumoniae*; PSA, *Pseudomonas aeruginosa*; ACN, *Acinetobacter baumannii*; AER, *Aeromonas veronii*; STM, *Stenotrophomonas maltophilia*; PSS, *Pseudomonas species*; ACP, *Acinetobacter pitti*; EMP, *Empedobacter falsenii*; ENT, *Enterobacter hormaechi* (base map adapted from "Lebanon_divisions.svg," created by Crates, under the Creative Commons Attribution-ShareAlike 3.0 License, https://creativecommons.org/licenses/by-sa/3.0/deed.en; modifications were made to the map using Microsoft Excel and PowerPoint).

The distribution of isolates harboring carbapenem resistance genes across the nine provinces is shown in Fig. 4 and Table S5. The highest occurrence of carbapenem resistance genes was found in Akkar and Mount Lebanon with 25% (22/87) and 23% (20/87), respectively, and the lowest in Keserwan-Jbeil and Beqaa with 4% (3/87) and 5% (4/87), respectively. Moreover, *bla*_NDM-5_ was predominant in all provinces, unlike *bla*_NDM-1_, which was only detected in three provinces. Co-expression of *bla*_NDM-5_ and *bla*_OXA-1_ was observed in Akkar, Baalbek, and Nabatieh. Carbapenemases such as *bla*_NDM-5_, *bla*_OXA-181_, and *bla*_POM-1_ were concentrated in Mount Lebanon. *bla*_POM-1_ was prevalent in six provinces, while *bla*_POM-2_ was exclusively detected in Baalbek-Hermel. Notably, *bla*_OXA-23_ was located mainly in Akkar. Despite its low prevalence, *bla*_OXA-72_ originated from one isolate in Mount Lebanon. Furthermore, *S. maltophilia* carrying *bla*_L1_ was present in three provinces, while *cphA3* and *bla*_EBR-4 2_ were isolated only in Mount Lebanon. The MexAB-OprM gene encoding the efflux pump was detected in three provinces and co-expressed with *bla*_IMP-1_ in Beirut.

## DISCUSSION

This study investigated the prevalence of critical carbapenem-resistant bacteria isolated from diverse districts across Lebanon. Our findings emphasized multiple sources of resistant Gram-negative bacteria that belong to clinically relevant STs that harbor various genes and plasmids encoding resistance to carbapenem.

These findings were consistent with previous studies done in Lebanon, as these microorganisms were highly detected in sewage and water samples. A nationwide study on water quality in Lebanon’s rivers revealed that approximately 46% of the recovered *E. coli* isolates were MDR (17). This is due to the discharge of untreated wastewater into the aquatic environment and the lack of connections to sewer networks (17). This highlights the role of water pollution as a major contributor to the environmental reservoirs of resistance genes in Lebanon. Another important factor influencing antimicrobial resistance patterns in the Lebanese environment is the intensive agricultural practices, including the misuse of antibiotics in poultry farming. As demonstrated by Shaib et al. in 2018, MDR *E. coli* was isolated from poultry in Lebanese broiler farms (18). Moreover, the variation in the distribution of isolates harboring carbapenem resistance genes across the nine provinces is asymmetrical, ranging from 4% in Keserwan-Jbeil to 25% in Akkar. Undoubtedly, several socioeconomic and demographic factors contribute to this variation. Interestingly, the capital Beirut, which is the most densely populated province, had a relatively low percentage of carbapenem-resistant isolates recovered (8%), while Akkar, which is less densely populated, had the highest percentage (25%). This indicates that, rather than population density, the variation in the quality of infrastructure and services provided across provinces contributes greatly to the variation in the occurrence of carbapenem-resistant isolates. Together, these factors create interconnected pathways for the spread of resistant bacteria, underscoring the need for integrated surveillance and improved wastewater management to limit the spread of resistant bacteria in the environment.

Remarkably, carbapenem-resistant *E. coli* was also detected in wild animals from the North province. To our knowledge, this is the first detection of carbapenem-resistant *E. coli* from wild animals in Lebanon, as previously conducted research was mainly focused on the spread of resistant bacteria in domestic animals and poultry. The detection of carbapenem-resistant *E. coli* in wild animals in Lebanon is particularly noteworthy, as it marks a significant expansion of the known reservoirs of these resistant pathogens. Thus, wild animals could facilitate the spread of carbapenem-resistant bacteria across ecological and geographical boundaries. This finding underscores the potential for zoonotic transmission of carbapenem resistance, a pressing public health concern given the limited treatment options available for such infections. It also raises questions about the human-derived factors contributing to the dissemination of these resistant strains into wildlife populations, such as contamination of water or food sources.

In our study, MLST revealed that recovered carbapenem-resistant *E. coli* belonged to seven main STs. The most prevalent ST was ST361, followed by ST405 and ST167; these were considered the most prevalent in clinical settings, representing international high-risk clones (19). Among others, *E. coli-*ST405 had been reported in hospitalized patients in different countries (20). In our study, ST405 was isolated from sewage and water (drinking, household, irrigation, and seawater) samples and was previously recovered by different studies from non-wild animals (21). Interestingly, our study confirmed the widespread presence of ST361, as it was detected in sewage, water (drinking and sea water), and soil samples. As for the recovered *K. pneumoniae* from sewage and seawater samples, they belonged to three STs. Worldwide, ST15 and ST147 were recognized as high-risk international clones associated with outbreaks and were commonly isolated from hospitalized patients (22, 23). Consistent with our study, *K. pneumoniae*-ST15 was isolated from river water and wastewater in China (24).

Furthermore, ST15, along with ST147, was detected in companion birds, highlighting the possible transmission of critical pathogens within a One Health perspective (22). Screening the entire set of 48 CRE isolates revealed various resistance genes, among which we focused on carbapenemases. Despite being reported elsewhere in environmental *E. coli* isolates (25, 26), no *E. coli* harboring *bla*_NDM-1_ were isolated in our study. However, *bla*_NDM-5_ was the most detected carbapenemase in almost all *E. coli* isolates and conferred greater resistance to extended-spectrum cephalosporins and carbapenems (27). *bla*_NDM-5_ was first detected in 2011 in an *E. coli*-infected patient in the United Kingdom (UK). Our findings corroborate those of multiple studies indicating that *bla*_NDM-5_-producing Enterobacterales had been recovered from several sources, including food, livestock, companion animals, wildlife, and the environment (28). It is worth mentioning that the first detected *bla*_NDM-5_ in the UK (27) belonged to ST648, similar to the one isolated in our study from an otter in North Lebanon.

Screening the genome of the four carbapenem-resistant *K. pneumoniae* isolates revealed the presence of *bla*_NDM-1_, *bla*_NDM-5_, and *bla*_OXA-181_. Our results align with prior data that demonstrated the occurrence of *Klebsiella* spp. isolates harboring *bla*_NDM_ and *bla*_OXA-48_-like genes in Lebanon (15). Worldwide, many studies have associated *bla*_NDM-1_-positive *K. pneumoniae* with infections in adults and neonates (29). Moreover, *bla*_NDM_-positive *K. pneumoniae* isolates of ST147 found in sewage and water samples were reported in multiple countries from human sources (30). Furthermore, *bla*_OXA-181_, a *bla*_OXA-48_ variant, was first reported in India in 2007 and is present mainly in *K. pneumoniae* and *E. coli* (31). In agreement with our study, *bla*_OXA-181_ co-expressed with *bla*_NDM-5_ in *K. pneumoniae* was reported in Lebanon, albeit from clinical isolates in North Lebanon (15).

The frequent detection of CRE in clinical and environmental settings could reveal mobilization through horizontal gene transfer, such as plasmids (32). The incompatibility group of plasmids was the major group detected in almost all isolated Enterobacterales. IncX3 has been shown to play a major role in disseminating ARGs among Enterobacterales in both humans and animals and was known to harbor *bla*_NDM-1_, *bla*_NDM-5_, and *bla*_OXA-181_ (31, 33). In addition, IncF plasmids harboring *bla*_NDM-5_ were detected in *E. coli* isolates and were widely spread among hospitalized patients in England (34). Zeng et al. highlighted that about 50% of the *bla*_NDM_-harboring plasmids of *K. pneumoniae* were IncF plasmids, with IncFIB(pNDM-Mar) and IncHI1B(pNDM-MAR) being the most dominant (35).

Another key finding in this study was the isolation of MDR ST2 *A. baumannii* from drinking and usage water samples, which poses a great threat to human health. Further analysis revealed the presence of an acquired class D oxacillinase, namely *bla*_OXA-23_. In line with these results, studies on AMR in hospital settings shed light on the prevalence of CRAB in the country, with *bla*_OXA-23_ being the most commonly detected gene (36). In their review, Sleiman et al*.* reported that recently about 82% of *A. baumannii* isolates collected from Lebanese hospitals were CRAB carrying *bla*_OXA-23_ (14). Several studies also noted the presence of *A. baumannii* in extra-human reservoirs. As an example, MDR ST2 *A. baumannii* was recovered from non-human sources in different countries (37). In Lebanon, *bla*_OXA-23_ was detected in *A. baumannii* isolated from livestock and poultry (12).

In this study, we isolated carbapenem-resistant *P. aeruginosa* harboring MexAB-OprM as the main mechanism for carbapenem resistance and identified three STs with ST1182 as the most prevalent. Lee et al. highlighted ST1182, ST224, and ST357 as critical for their involvement in ARG dissemination within different species (38). In Lebanon, data showed that about 40%–97.1% of *P. aeruginosa* infections were due to carbapenem-resistant isolates that encompassed carbapenem-hydrolyzing enzymes and non-enzymatic mechanisms such as alteration of the outer membrane porin protein and overexpression of efflux pumps (39). Consistent with our results, literature showed that among the diverse resistance mechanisms, multidrug efflux pump MexAB-OprM exhibits greater prominence and contributes to MDR in *P. aeruginosa* and to their resistance against carbapenem (39). In addition to the expression of MexAB-OprM, *P. aeruginosa* ST357 carried *bla*_IMP-1_, which matches the previous literature associating ST357 *P. aeruginosa* with *bla*_IMP_ expression (40).

Other carbapenem-resistant non*-*Enterobacterales were detected in this study. Significantly, all *S. maltophilia* isolates expressed MBLs, *bla*_L1_, or *bla*_L2_*,* conferring intrinsic resistance to a wide range of antibiotics, including carbapenems (16). *S. maltophilia,* an emerging pathogen as described by the WHO, is associated with systemic infections with about 69% mortality rate and has been recovered from various hospital environments (41). Interestingly, two *C. gilardii* isolates recovered from water samples expressed the *mcr5.1* gene that encodes resistance to colistin. In agreement with our study, Cherak et al. reported the first detection of the *mcr-5.1* gene in *C. gilardii* isolated from well water in Algeria (42). Cupriavidus is considered an emerging pathogen that was found in clinical specimens and environmental sources and was also associated with several infections (42).

The occurrence of carbapenem-resistant pathogens in animals and the environment in Lebanon poses a growing threat. Extra-hospital settings emerge as significant reservoirs, facilitating resistance transmission to humans and endangering public health. To address this issue, critical actions should be taken, including enhanced wastewater management and sewage treatment to reduce the release of antimicrobial-resistant bacteria and genes into natural ecosystems. Second, implementing nationwide surveillance programs that monitor antimicrobial resistance in environmental samples, including wildlife and water sources, especially using next-generation sequencing. Such programs would incorporate a “One Health” approach to assess the interdependence of human, animal, and environmental health. Finally, raising awareness about the risks of antibiotic misuse in agriculture and ensuring strict adherence to antimicrobial stewardship policies would help reduce the introduction of resistance genes into the environment.

## MATERIALS AND METHODS

### Sample collection

Multiple sample collection criteria were considered in order to achieve a diversity that ensures representative and reliable findings. Samples were collected from random locations within urban, rural, and natural areas in order to attain location diversity. Moreover, a variety of sample types were considered, such as water, soil, and animals. Furthermore, all samples were collected over a duration that covered multiple seasons. In addition, animal and water samples were collected from various types and sources.

Up to 250 samples from different locations across Lebanon were collected and divided as follows: 152 water samples, 60 sewage samples, 29 animal samples, and 9 soil samples. All samples were received from June 2022 until September 2023. All samples were transported aseptically under refrigeration conditions (4°C) to the Experimental Pathology, Immunology and Microbiology Research Laboratory at the American University of Beirut for processing, where they were stored at –20°C until further analysis within 24 h after collection.

#### Collection of water samples

A total of 152 water samples were collected as shown in Table S3. Among those, 12 samples came from the marine environment, which included four spring water samples collected from Byblos (Afqa), three fountain water samples in Akkar, two sea water samples collected from Mount Lebanon at Antelias area, and one river sample collected from each of South (Litani), Beqaa (Assi), and Mount Lebanon (Nahr Ibrahim). The other 141 samples were gathered as follows: 53 from drinking water, 29 samples came from irrigation canals, 26 using water sources, 13 from storage tanks, and 19 from house tap water. All samples were collected in 1 L sterile bottles, which were previously rinsed with distilled water and autoclaved at 121°C for 15 min.

#### Collection of sewage samples

A total of 60 sewage samples from different locations: Akkar (10), Mount Lebanon (9), Beirut (8), Beqaa (8), Baalbek-Hermel (7), South (7), Nabatieh (6), and North (5) were collected as shown in Fig. 1. The samples from raw untreated wastewater from each sampling site were collected in 1 L sterile bottles which were previously rinsed with distilled water and autoclaved at 121°C for 15 min. Because our country lacks functional wastewater treatment plants, all samples were mainly obtained from wastewater influents discharged into the ocean, and from multiple municipalities, households, refugee camps, and businesses’ wastewater. Safety guidelines and precautions were observed throughout the processes of sample collection, transportation, analysis, and disposal.

#### Collection of animal samples

A total of 29 animal samples were provided by the Lebanese Wildlife Team, collected during their field work from 26 living and three dead animals (Table S4). The animals belonged to three main groups, divided as follows: three invertebrates, eight mammals, and 18 reptiles, as represented in the supplementary table. Different swab types were collected from the wild-caught or patient-admitted animals and distributed as follows: 13 body swabs and eight cloacal swabs. For cloacal and body swabbing, we used an invasive sterile collection swab maintained in a Cary-Blair transport medium tube (BOENMED Boen Healthcare Co., Ltd., Suzhou, China). For mammals, eight faecal specimens were deposited in a 15 mL sterile Falcon tube (Corning, New York, USA).

#### Collection of soil samples

The soil samples were collected from an agricultural field, mainly Beddawi Gardens Farm in the North. It is a seed farm for Socio-Economic Action Collective, an agricultural collective that takes the lead in the struggle against conventional agriculture. The samples come from a range of soil types on the farm. The nine soil samples were collected from aromatic crops (moringa, malifa, lavender), kale, beans, wild oats, and wheat crops. The soil samples were obtained from nine different locations at a depth of 0–20 cm using a sterile metal spatula and transferred to transparent polyethylene bags.

### Isolation and purification of carbapenem-resistant Gram-negative bacteria

Environmental samples were enriched with 6 mL autoclaved peptone water. The mixture was incubated overnight at 35°C ± 2°C on a shaker at 160 rpm. After 18–24 h, an aliquot (20–30 µL) was cultured on MacConkey (Neogen, Michigan, USA) agar plate supplemented with 2 mg/L meropenem (Sigma-Aldrich, Missouri, USA) or CHROMagar mSuperCARBA base (CHROMagar, Paris, France). Meropenem is more effective than imipenem against carbapenem-resistant Gram-negative bacteria, including *Pseudomonas* and ESBL-producing strains, due to its broader spectrum of activity. The plates were checked for growth after 24, 48, and 72 h. If growth was observed, each colony with different morphology was sub-cultured on separate MacConkey agar plates until pure cultures were observed. Few well-isolated colonies were added to 3–4 mL Luria Bertani broth (Bio-Rad, California, USA) in polystyrene tubes and incubated overnight. After 24 h, 600 µL of bacterial mixture was transferred to 50% glycerol tubes and stored at −80°C.

### Gram staining

Gram staining was adapted from bacteriologist Hans Christian Gram (10.1002/9780471729259.mca03cs00).

### Oxidase test

The oxidase test was performed using the oxidase disc (70439-50DISKS-F) from Millipore according to the manufacturer’s protocol. A well-isolated colony was spread on an oxidase disc using a loop. The appearance of purple color indicates a positive oxidase test, and the appearance of pink or no color indicates a negative oxidase test.

### Analytical profile index 20E test

The analytical profile index (API) 20E test was performed according to the manufacturer’s protocol (BioMérieux, 69280, Marcy l’étoile, France), and the organism was identified to the species level using API web software.

### Antimicrobial susceptibility testing

An antimicrobial susceptibility test was performed using the Kirby-Bauer disk diffusion method. The following antibiotic discs were used: meropenem (MEM) 10 µg, ciprofloxacin (CIP) 5 µg, gentamicin (GMN) 10 µg, ceftazidime (CAZ) 10 µg, and aztreonam (ATM) 30 µg. The use of meropenem in both the isolation and susceptibility testing steps ensures consistency in assessing resistance. The initial use of meropenem selects for bacteria that can grow in its presence, while the subsequent AST quantifies the level of resistance. This approach verifies that the isolates are indeed resistant and determines the extent of their resistance. For each isolate, a suspension of 0.5 McFarland in Mueller-Hinton (MH) broth was prepared and spread on an MH agar plate using a sterile swab to produce confluent growth. After 10 min, the five antibiotic discs were placed diversely on the plate. The plate was left in the incubator at 35°C ± 2°C for 18 to 24 h. After incubation, the diameter of the zone of inhibition was measured and recorded. The results were analyzed according to the CLSI M100Ed33 guidelines (16). Another antimicrobial susceptibility test was performed using the BioMérieux Vitek 2 compact system using GN93 cards according to the manufacturer’s protocol.

### DNA extraction

Bacterial genomic DNA was extracted from bacterial isolates cultured on MacConkey agar using the Quick-DNA Fungal/Bacterial Miniprep kit, then DNA was purified using the Genomic DNA Clean & Concentrator kit (Zymo Research, Irvine, CA) according to the manufacturer’s protocols.

### Whole-genome sequencing

#### Short-read sequencing

Short-read whole-genome sequencing was carried out using the Illumina platform. The Illumina DNA prep kit, along with the IDT for Illumina DNA UD indexes (Illumina, San Diego, CA) was used to prepare DNA libraries as per the manufacturer’s protocol. Then, library concentrations were measured using the Qubit dsDNA High Sensitivity assay kit (Thermo Fisher Scientific, Waltham, MA) on a Qubit 4 fluorometer (Thermo Fisher Scientific, Waltham, MA). After pooling, denaturing, and diluting DNA libraries, PhiX Control v3 (Illumina, San Diego, CA) was added, and then the pooled library was sequenced using a MiSeq V2 Reagent Kit (500 cycles) on an Illumina MiSeq platform (Illumina, San Diego, CA) for 250 × 2 cycles for a coverage of 100×.

### Bioinformatics analysis

Most of the bioinformatics analysis was done using tools offered by the Usegalaxy platform (https://usegalaxy.org/). The quality of the files was checked using FastQC (v.0.74) (PMID: 24834071), and trimming of the reads was performed accordingly using Trimmomatic (v.1.2.14) (PMID: 24695404), after which the assembly of the paired reads was performed using Unicycler (v.0.5.1) (PMID: 28594827). Antimicrobial resistance genes were acquired through CARD (https://card.mcmaster.ca/) and AMRFinder (PMID: 34135355) (40). Sequence types were identified using MLST (PMID: 22238442) on UseGalaxy. Plasmid analysis was conducted using PlasmidFinder (PMID: 24777092) (43). The isolate identification was performed using BLASTN (PMID: 18440982) (44) with the default pipeline parameters (40). Plasmid analysis was conducted using PlasmidFinder (PMID: 24777092) (43). The isolate identification was performed using BLASTN (PMID: 18440982) (44) with the default pipeline parameters.

## Data Availability

The data sets generated during the current study are available in the NCBI repository under BioProject number PRJNA613441, with the accession numbers provided in Data set S1 in the supplemental material.

## SUPPLEMENTAL MATERIAL

Data Set S1 aem.01932-24-s0001.xlsx Accession numbers.

Supplemental tables aem.01932-24-s0002.docx Tables S1 to S5.

ASM does not own the copyrights to Supplemental Material that may be linked to, or accessed through, an article. The authors have granted ASM a non-exclusive, world-wide license to publish the Supplemental Material files. Please contact the corresponding author directly for reuse.
