# Supplementary material for: Nationwide surveillance of carbapenem-resistant Gram-negative pathogens in the Lebanese environment
Source: Appl Environ Microbiol. 2025 Jun 10;91(7):e01932-24. doi: 10.1128/aem.01932-24 (PMC12285220; doi:10.1128/aem.01932-24)
Supplement: Supplemental tables — Tables S1 to S5. [file aem.01932-24-s0002.docx]

| Isolate Data | | | | | | Plasmids | |
| --- | --- | --- | --- | --- | --- | --- | --- |
| Year of Isolation | Isolate | Organism | Sampling Site | Sampling type | ST | Inc group | Col plasmids |
| 2022 | ECOL_194 | *Escherichia coli* | Akkar | Sewage | 361 | IncFIA, IncFII, IncI (Gamma), IncY | - |
| 2022 | ECOL_195 | *Escherichia coli* | Akkar | Sewage | 361 | IncFIA, IncFII, IncI (Gamma), IncY | - |
| 2022 | ECOL_196 | *Escherichia coli* | Akkar | Water | 405 | IncFIA, IncFIB(AP001918), IncFIl, IncI2(Delta) | Col(MG828) |
| 2022 | ECOL_197 | *Escherichia coli* | Akkar | Sewage | 405 | IncFIA, IncFII, IncFIB(AP001918) | Col(MG828) |
| 2022 | ECOL_198 | *Escherichia coli* | North | Animal | 648 | IncFIA, IncFIB (AP001918), IncFIl (pRSB107) | - |
| 2022 | ECOL_199 | *Escherichia coli* | Beqaa | Sewage | 405 | IncFIA, IncFII(pRSB107) | Col15, Col(BS512) |
| 2023 | ECOL_200 | *Escherichia coli* | Beqaa | Sewage | 361 | IncFIA, IncFII, IncI1-I(Alpha), IncY | - |
| 2022 | ECOL_201 | *Escherichia coli* | Baalbek-Hermel | Sewage | 405 | IncFIA, IncFIB(AP001918), IncFII | Col(MG828) |
| 2022 | ECOL_202 | *Escherichia coli* | Baalbek-Hermel | Sewage | 405 | IncFIA, IncFIB(AP001918), IncFII | Col(MG828) |
| 2022 | ECOL_203 | *Escherichia coli* | Baalbek-Hermel | Water | 405 | IncFIA, IncFIB(AP001918), IncI1-I(Alpha), IncFII | Col(MG828) |
| 2022 | ECOL_205 | *Escherichia coli* | Akkar | Sewage | 361 | IncFIA, IncFII, IncI (Gamma), IncY | - |
| 2022 | ECOL_206 | *Escherichia coli* | North | Sewage | 405 | IncFIA, IncFII, IncI1-I(Alpha), p0111 | Col (MG828) |
| 2022 | ECOL_207 | *Escherichia coli* | Akkar | Sewage | 405 | IncFIA, IncFII, IncI1-I(Alpha), p0111 | - |
| 2022 | ECOL_208 | *Escherichia coli* | Akkar | Sewage | 2083 | IncFIB(pB171), IncFII, IncI(Gamma) | Col(MG828), Col (pHAD28) |
| 2022 | ECOL_209 | *Escherichia coli* | Nabatieh | Sewage | 361 | IncFIA, IncFII, IncI(Gamma), IncY | - |
| 2022 | ECOL_210 | *Escherichia coli* | South | Sewage | 167 | IncFIA, IncFII, IncX2 | Col(BS512), Col(pHAD28) |
| 2022 | ECOL_211 | *Escherichia coli* | Nabatieh | Sewage | 405 | IncFIA, IncFIB(AP001918), IncI1-I(Alpha), IncFII | Col(MG828) |
| 2022 | ECOL_212 | *Escherichia coli* | South | Sewage | - | IncFIA, IncFII, IncI(Gamma),IncY | - |
| 2022 | ECOL_213 | *Escherichia coli* | South | Water | 2083 | IncFIB(pB171), IncI(Gamma), IncFII | Col(MG828), Col(pHAD28) |
| 2022 | ECOL_216 | *Escherichia coli* | Beirut | Sewage | 361 | IncFIA, IncFII, IncY, Incl1-I(Alpha) | Col156 |
| 2023 | ECOL_217 | *Escherichia coli* | Beirut | Sewage | 361 | IncFIA, IncFII, Incl1-l(Alpha), IncY | - |
| 2022 | ECOL_218 | *Escherichia coli* | Mount Lebanon | Sewage | 167 | IncFIA, IncFII | Col156, Col(BS512) |
| 2022 | ECOL_219 | *Escherichia coli* | Mount Lebanon | Sewage | 361 | IncFIA, IncFII, IncI(Gamma), IncI2, IncY | - |
| 2022 | ECOL_220 | *Escherichia coli* | Mount Lebanon | Soil | 361 | IncFIA, IncFII, IncI(Gamma), IncY | - |
| 2022 | ECOL_221 | *Escherichia coli* | Mount Lebanon | Sewage | 90 | IncX3 | - |
| 2022 | ECOL_222 | *Escherichia coli* | Mount Lebanon | Water | 10 | IncFIB(AP001918), IncFII, IncQ1 | - |
| 2022 | ECOL_223 | *Escherichia coli* | Baalbek-Hermel | Water | 361 | IncFIA, IncFII, IncY | - |
| 2022 | ECOL_225 | *Escherichia coli* | South | Sewage | 167 | IncFIA, IncFII, IncX1 | Col(BS512), Col(pHAD28) |
| 2022 | ECOL_226 | *Escherichia coli* | Nabatieh | Sewage | 167 | IncFIA, IncFII, IncX1 | Col(BS512), Col(pHAD28),Col 440I |
| 2023 | ECOL_228 | *Escherichia coli* | Mount Lebanon | Water | 405 | IncFIA, IncFII, p0111 | - |
| 2023 | ECOL_229 | *Escherichia coli* | Mount Lebanon | Water | 361 | IncFIA, IncFII, IncI(Gamma), IncY | - |
| 2023 | ECOL_230 | *Escherichia coli* | Mount Lebanon | Water | 361 | IncFIA, IncFII, IncI(Gamma), IncY | - |
| 2023 | ECOL_231 | *Escherichia coli* | Mount Lebanon | Water | 405 | IncFIA, IncFIB(AP001918), IncFII, | Col(MG828), Col(IRGK) |
| 2023 | ECOL_232 | *Escherichia coli* | Mount Lebanon | Water | 167 | IncFIA, IncFII, IncX1 | Col(BS512), Col(pHAD28) |
| 2022 | ECOL_233 | *Escherichia coli* | Akkar | Water | 361 | IncFIA, IncFII, IncI(Gamma), IncY | - |
| 2022 | ECOL_234 | *Escherichia coli* | Akkar | Water | 405 | IncFIA, IncFII, IncI1-I(Alpha), p0111 | - |
| 2022 | ECOL_235 | *Escherichia coli* | Akkar | Water | 2083 | IncFIB(pB171), IncI(Gamma), IncFII | Col(MG828), Col(pHAD28) |
| 2022 | ECOL_236 | *Escherichia coli* | South | Sewage | 361 | IncFIA, IncFII, IncI(Gamma), IncY | - |
| 2022 | ECOL_237 | *Escherichia coli* | North | Sewage | 405 | IncFIA, IncFII, IncI1-I(Alpha), p0111 | - |
| 2023 | ECOL_238 | *Escherichia coli* | Mount Lebanon | Sewage | 10 | IncFIB(AP001918), IncFII, IncQ1 | - |
| 2022 | ECOL_239 | *Escherichia coli* | Baalbek-Hermel | Sewage | 167 | IncFIA, IncFII, IncY | - |
| 2023 | ECOL_240 | *Escherichia coli* | Beirut | Sewage | 361 | IncFIA, IncFII, IncFII(pSE11), IncY | - |
| 2023 | ECOL_241 | *Escherichia coli* | Beirut | Sewage | 167 | IncFIA, IncFIB(AP001918), IncFII, IncFI(Gamma), IncI2(Delta) | Col156 |
| 2023 | KLB_111 | *Klebsiella pneumoniae* | Mount Lebanon | Water | 16 | IncFIA(HI1), IncFII, IncR, IncX3 | Col (pHAD28), Col440II, ColKP3 |
| 2023 | KLB_112 | *Klebsiella pneumoniae* | Nabatieh | Sewage | 147 | IncFIB(pNDM-Mar), IncHI1B(pNDM-MAR), IncFIB(pQil), IncFIB(pKPHS1) | Col(pHAD28) |
| 2023 | KLB_113 | *Klebsiella pneumoniae* | Beirut | Sewage | 15 | IncFIB(K), IncFIB(pB171), IncFII(k), IncFII(Yp), IncX4 | - |
| 2023 | KLB_114 | *Klebsiella pneumoniae* | Mount Lebanon | Water | 147 | IncFIB(pQil), IncR | Col(pHAD28) |
| 2023 | PSA_702 | *Pseudomonas aeruginosa* | Mount Lebanon | Sewage | 1182 | IncFIA | Col(BS512), Col(MG828) |

Supplementary Table 1- The detected plasmids in different isolates.

| Isolate Data | | | | | | Resistance genes | Plasmids | |
| --- | --- | --- | --- | --- | --- | --- | --- | --- |
| Year of Isolation | Isolate | Organism | Sampling Site | Sampling type | ST | Carbapenem | Inc group | Col plasmids |
| 2022 | ACP_001 | Acinetobacter pitti | Mount Lebanon | Soil | 1687 | OXA-72 | - | - |
| 2022 | AER_001 | Aeromonas veronii | Beqaa | Sewage | 425* | cphA3 | - | |
| 2023 | AER_002 | Aeromonas veronii | Mount Lebanon | Water | 2182 | cphA3 | IncU | - |
| 2022 | CIT_002 | Citrobacter fruendii | Mount Lebanon | Animal | 234 | - | - | |
| 2022 | CIT_003 | Citrobacter fruendii | Mount Lebanon | Animal | 247* | - |  |  |
| 2022 | CUP_001 | Cupriavidus gilardii | Baalbek-Hermel | Water | - | - |  |  |
| 2022 | CUP_002 | Cupriavidus gilardii | Akkar | Water | - | - |  |  |
| 2023 | CUP_003 | Cupriavidus gilardii | North | Soil | - | - |  |  |
| 2022 | CUP_004 | Cupriavidus gilardii | North | Soil | - | - |  |  |
| 2023 | CUP_005 | Cupriavidus gilardii | Akkar | Water | - | - |  |  |
| 2022 | EMP_001 | Empedobacter falsenii | Akkar | Water | - | EBR-4 |  |  |
| 2023 | ENT_05 | Enterobacter hormaechi | Beqaa | Sewage | - | NDM-1 | IncFII(pKPX1), IncF(repB(R1701)) | Col(pHAD28) |
| 2023 | PAN_001 | Pannonibacter phragmitetus | Mount Lebanon | Sewage | - | - | - | |
| 2022 | PSS_012 | Pseudomonas fluorescens | Mount Lebanon | Water | 5* | - |  |  |
| 2022 | PSS_033 | Pseudomonas otitidis | Akkar | Water | - | POM-1 |  |  |
| 2022 | PSS_034 | Pseudomonas otitidis | South | Water | - | POM-1 |  |  |
| 2022 | PSS_035 | Pseudomonas otitidis | South | Water | - | POM-1 |  |  |
| 2022 | PSS_036 | Pseudomonas otitidis | South | Water | - | POM-1 |  |  |
| 2022 | PSS_037 | Pseudomonas otitidis | South | Water | - | POM-1 |  |  |
| 2023 | PSS_038 | Pseudomonas otitidis | Mount Lebanon | Sewage | - | POM-1 |  |  |
| 2023 | PSS_039 | Pseudomonas otitidis | Beqaa | Sewage | - | POM-1 |  |  |
| 2022 | PSS_040 | Pseudomonas otitidis | Baalbek-Hermel | Sewage | - | POM-2 |  |  |
| 2022 | PSS_041 | Pseudomonas otitidis | Akkar | Water | - | POM-1 |  |  |
| 2023 | PSS_001 | Pseudomonas putida | Beqaa | Sewage | - | - |  |  |
| 2022 | PSS_002 | Pseudomonas putida | Akkar | Water | 200* | - |  |  |
| 2022 | PSS_003 | Pseudomonas putida | Akkar | Water | 97* | - |  |  |
| 2022 | PSS_004 | Pseudomonas putida | Akkar | Water | - | - |  |  |
| 2022 | PSS_005 | Pseudomonas putida | Akkar | Water | 14* | - |  |  |
| 2022 | PSS_006 | Pseudomonas putida | North | Soil | 134* | - |  |  |
| 2022 | PSS_007 | Pseudomonas putida | North | Soil | 134* | - |  |  |
| 2022 | PSS_008 | Pseudomonas putida | North | Animal | 222* | - |  |  |
| 2022 | PSS_009 | Pseudomonas putida | North | Animal | 230* | - |  |  |
| 2023 | PSS_010 | Pseudomonas putida | Mount Lebanon | Sewage | - | - |  |  |
| 2023 | PSS_011 | Pseudomonas putida | Mount Lebanon | Sewage | 32* | - |  |  |
| 2022 | PSS_013 | Pseudomonas putida | Mount Lebanon | Sewage | 230* | - |  |  |
| 2022 | PSS_014 | Pseudomonas putida | Mount Lebanon | Water | 200* | - |  |  |
| 2023 | PSS_015 | Pseudomonas putida | Mount Lebanon | Sewage | 137 | - |  |  |
| 2023 | PSS_016 | Pseudomonas putida | Mount Lebanon | Water | 193* | - |  |  |
| 2023 | PSS_017 | Pseudomonas putida | Mount Lebanon | Water | 97* | - |  |  |
| 2022 | PSS_018 | Pseudomonas putida | Mount Lebanon | Animal | 106* | - |  |  |
| 2022 | PSS_019 | Pseudomonas putida | Mount Lebanon | Animal | 14* | - |  |  |
| 2022 | PSS_020 | Pseudomonas putida | Mount Lebanon | Animal | 193* | - |  |  |
| 2023 | PSS_021 | Pseudomonas putida | Mount Lebanon | Water | 69* | - |  |  |
| 2022 | PSS_022 | Pseudomonas putida | Akkar | Water | 17* | - |  |  |
| 2023 | PSS_023 | Pseudomonas putida | Mount Lebanon | Water | 193* | - |  |  |
| 2023 | PSS_024 | Pseudomonas putida | South | Sewage | 136* | - |  |  |
| 2023 | PSS_025 | Pseudomonas putida | South | Water | 14* | - |  |  |
| 2023 | PSS_026 | Pseudomonas putida | Mount Lebanon | Sewage | 185* | - |  |  |
| 2023 | PSS_027 | Pseudomonas putida | Mount Lebanon | Water | 262* | - |  |  |
| 2023 | PSS_028 | Pseudomonas putida | Baalbek-Hermel | Water | - | - |  |  |
| 2023 | PSS_029 | Pseudomonas stutzeri | Mount Lebanon | Sewage | - | PST-2 |  |  |
| 2022 | PSS_032 | Pseudomonas stutzeri | Akkar | Water | - | PST-2 |  |  |
| 2023 | PSS_030 | Pseudomonas species | Mount Lebanon | Sewage | - | PST-2 |  |  |
| 2023 | PSS_031 | Pseudomonas species | Mount Lebanon | Water | - | - |  |  |
| 2023 | STM_004 | Stenotrophomonas maltophilia | Baalbek-Hermel | Water | 909* | L1 |  |  |
| 2022 | STM_005 | Stenotrophomonas maltophilia | Baalbeck-Hermel | Water | 566* | L2 |  |  |
| 2022 | STM_006 | Stenotrophomonas maltophilia | Mount Lebanon | Soil | 10126* | L1 |  |  |
| 2022 | STM_007 | Stenotrophomonas maltophilia | Mount Lebanon | Sewage | 874* | L1 |  |  |
| 2022 | STM_008 | Stenotrophomonas maltophilia | Mount Lebanon | Water | 138* | L1 |  |  |
| 2022 | STM_009 | Stenotrophomonas maltophilia | Mount Lebanon | Animal | 748* | L1 |  |  |
| 2022 | STM_010 | Stenotrophomonas maltophilia | Akkar | Water | 605* | L1 |  |  |
| 2023 | STM_011 | Stenotrophomonas maltophilia | Mount Lebanon | Sewage | 599* | L1 |  |  |
| 2022 | STM_012 | Stenotrophomonas maltophilia | Akkar | Water | - | L1 |  |  |

Supplementary Table 2- Other species with carbapenem-resistance genes and plasmids

| Province/ Water Type | Drinking | Irrigation | Using | Tank | Tap | River | Sea | Fountain | Spring |
| --- | --- | --- | --- | --- | --- | --- | --- | --- | --- |
| Akkar | 25 | 12 | 11 | 8 | 6 |  |  | 3 |  |
| Baalbek-Hermel | 10 | 6 | 2 | 4 | 3 |  |  |  |  |
| Beqaa |  | 5 | 1 | 1 | 5 | 1 |  |  |  |
| South | 9 | 1 | 1 |  |  | 1 |  |  |  |
| Mount Lebanon | 7 | 5 | 11 |  | 5 | 1 | 2 |  |  |
| Keserwan-Jbeil | 2 |  |  |  |  |  |  |  | 4 |

Supplementary Table 3- The number and type of different water samples collected within the Lebanese provinces.

| Species common name | Species scientific name | Location | Type | Status | Sample type | Age | Sex |
| --- | --- | --- | --- | --- | --- | --- | --- |
| Bat Colony | N/A | Mount Lebanon | Wild-Caught | Alive | Fecal | UNK | UNK |
| Bat Colony | N/A | Mount Lebanon | Wild-Caught | Alive | Fecal | UNK | UNK |
| Bat Colony | N/A | Mount Lebanon | Wild-Caught | Alive | Fecal | UNK | UNK |
| Eurasian Otter | *Lutra lutra* | Akkar | Wild-Caught | Alive | Fecal | UNK | UNK |
| Eurasian Otter | *Lutra lutra* | Akkar | Wild-Caught | Alive | Fecal | UNK | UNK |
| Eurasian Otter | *Lutra lutra* | Akkar | Wild-Caught | Alive | Fecal | UNK | UNK |
| Fox/Hedgehog | N/A | Akkar | Wild-Caught | Alive | Fecal | UNK | UNK |
| Rodent | N/A | North | Wild-Caught | Alive | Fecal | UNK | UNK |
| Legless Lizard | *Pseudopus apodus levantinus* | Nabatiyeh | Wild-Caught | Alive | Cloaca Swab | Adult | Male |
| Schokari Sand Racer | *Psammophis schokari* | Nabatiyeh | Wild-Caught | Alive | Cloaca Swab | Adult | Male |
| Mediterranean Thin‑toed Gecko | *Mediodactylus orientalis* | Nabatiyeh | Wild-Caught | Alive | Body Swab | Juvenile | UNK |
| Snake-eyed Lizard | *Ophisops elegans* | Nabatiyeh | Wild-Caught | Alive | Cloaca Swab | Adult | Male |
| Palestinian Viper | *Daboia palaestinae* | Nabatiyeh | Wild-Caught | Dead | Body Swab | Adult | UNK |
| Black Whip Snake | *Dolichophis jugularis* | Nabatiyeh | Wild-Caught | Dead | Body Swab | Adult | UNK |
| Green Lizard | *Lacerta media* | Mount Lebanon | Wild-Caught | Alive | Cloaca Swab | Sub-adult | Male |
| Eastern Montpellier Snake | *Malpolon insgnitus* | Keserwan-Jbeil | Wild-Caught | Alive | Cloaca Swab | Young Adult | Male |
| Coin-marked Snake | *Hemorrhous nummifer* | Mount Lebanon | Patient Admission | Alive | Cloaca Swab | Young Adult | Female |
| Horny-scaled Agama | *Trapelus ruderatus* | Baalbek Hermel | Wild-Caught | Alive | Cloaca Swab | Adult | Male |
| Horny-scaled Agama | *Trapelus ruderatus* | Baalbek Hermel | Wild-Caught | Alive | Cloaca Swab | Adult | Male |
| Crowned Dwarf Racer | *Eirenis coronalla* | Baalbek Hermel | Wild-Caught | Alive | Body Swab | Juvenile | Male |
| Schneider's Skink | *Eumeces schniederi* | Baalbek Hermel | Wild-Caught | Alive | Body Swab | Juvenile | Male |
| Black Headed Cat Snake | *Telescopus nigriceps* | Baalbek Hermel | Wild-Caught | Alive | Body Swab | Adult | Female |
| Greek Tortoise | *Testudo graeca* | Akkar | Wild-Caught | Alive | Body Swab | Adult | UNK |
| Greek Tortoise | *Testudo graeca* | Akkar | Wild-Caught | Alive | Body Swab | Adult | UNK |
| Mediterranean Chameleon | *Chamaeleo chamaeleon* | North | Wild-Caught | Alive | Body Swab | Adult | UNK |
| Greek Tortoise | *Testudo graeca* | Akkar | Wild-Caught | Alive | Body Swab | Adult | UNK |
| Middle East Gold / Black Tarantula | *Chaetopelma olivaceum* | Keserwan-Jbeil | Wild-Caught | Alive | Body Swab | Sub-Adult | UNK |
| Death Stalker Scorpion | *Leiurus sp.* | Baalbek Hermel | Wild-Caught | Alive | Body Swab | Adult | UNK |
| Levantine freshwater crab | *Potamon potamios* | Akkar | Wild-Caught | Dead | Body Swab | Adult | UNK |

Supplementary Table 4 - Details on wild animal sample collection from various districts in Lebanon. N/A: not available, UNK: unknown

| Provinces | Fraction of isolates harboring carbapenem resistance genes | Percentage of isolates harboring carbapenem resistance genes |
| --- | --- | --- |
| Akkar | 22/87 | 25 % |
| Baalbek-Hermel | 8/87 | 9 % |
| beirut | 7/87 | 8 % |
| beqaa | 4/87 | 5 % |
| Keserwan-jbeil | 3/87 | 4 % |
| mount lebanon | 20/87 | 23 % |
| nabatieh | 5/87 | 6 % |
| north | 8/87 | 9 % |
| south | 10/87 | 11 % |

Supplementary Table 5 – Distribution of isolates harboring carbapenem resistance genes across the nine provinces.
